# Low calcium intake from diet and supplements in a group of Polish patients with SLE – an additional risk for osteoporotic fractures?

**DOI:** 10.3389/abp.2026.15808

**Published:** 2026-02-04

**Authors:** Jolanta Anna Dardzińska, Natalia Matysiak, Karolina Kułakowska, Marta Jaskólska, Sylwia Małgorzewicz, Alessio Molfino

**Affiliations:** 1 Department of Clinical Nutrition, Medical University of Gdansk, Gdańsk, Poland; 2 Independent Dietitians’ Team, University Clinical Centre, Gdańsk, Poland; 3 Department of Translational and Precision Medicine, Sapienza University of Rome, Rome, Italy; 4 Department of Rheumatology, Clinical Immunology, Geriatrics and Internal Medicine, Medical University of Gdansk, Gdańsk, Poland; 5 Department of Interdisciplinary Studies on WellBeing, Health and Environmental Sustainability (BeSSA) Sapienza University of Rome, Rome, Italy

**Keywords:** calcium intake, health awareness, osteoporosis, prevention, systemic lupus erythematosus

## Abstract

**Background:**

Patients with systemic lupus erythematosus (SLE) are at increased risk of osteoporosis. Despite this, the awareness of adequate dietary calcium intake and other prevention strategies is often underrecognized in clinical practice and guidelines.

**Methods:**

Dietary calcium intake was assessed using the ADOS-Ca questionnaire, validated for the Polish population, in SLE patients recruited both in person and online from various outpatient clinics. Data on vitamin D supplementation, awareness of osteoporosis risk, and preventive actions initiated by healthcare providers were also collected.

**Results:**

Median dietary calcium intake was 540 mg/day, below the recommended ≥1,000 mg or ≥1,200 mg (depending on age and sex). Only 25% of patients met these recommendations, including the subgroup of chronically taking glucocorticoids. Calcium supplementation was used by 31% of respondents on long-term glucocorticoid therapy. Regular vitamin D supplementation was reported by 79% of participants. Calcium and vitamin D intake and supplementation did not differ between patients treated and untreated with glucocorticoids. While 57% were aware of their elevated osteoporosis risk, only 32% had received guidance on preventive measures.

**Conclusion:**

Greater attention to osteoporosis prevention is needed among SLE patients, both from healthcare professionals and the patients themselves. The first and most fundamental step is optimizing calcium intake through diet and supplementation, which is particularly important in undergoing long-term glucocorticoid therapy.

## Introduction

Systemic lupus erythematosus [SLE] is an autoimmune disease with a prevalence in Europe ranging from 29 to 210 per 100,000 individuals (Barber et al., 2021) which allows it to be classified as a rare disease with a particular burden and challenges in healthcare (Cyske et al., 2025). Women are 6–10 times more likely to be affected than men, with the peak incidence occurring between the ages of 15 and 45 years (Fava and Petri, 2019). The increased prevalence of lupus in women of reproductive age suggests a role for estrogens in the pathogenesis of the disease (Barber et al., 2021).

In contrast to other organ-specific autoimmune diseases, the complex immune system disorders present in SLE lead to the development of a systemic inflammatory process (Dörner and Furie, 2019). Consequently, in addition to general symptoms such as fatigue, low-grade fever or fever, and weight loss, patients also exhibit multiple systemic and organ-specific symptoms. These include changes in the skin and mucous membranes, kidneys [lupus nephritis], musculoskeletal, respiratory, nervous, and cardiovascular systems. Hematologic and gastrointestinal disorders may also occur (Dörner and Furie, 2019). The disease course is characterized by periods of exacerbations and remissions. The primary treatment goal is to extend patient survival, prevent permanent organ damage, mini-mize adverse effects of therapy, and improve health-related quality of life (Muñoz-Grajales et al., 2023; Fanouriakis et al., 2024).

Among the numerous organ-related consequences of SLE, the increased risk of bone mass loss should not be overlooked (Orsolini et al., 2020). Numerous studies have confirmed that SLE patients exhibit a higher prevalence of reduced bone mineral density [BMD] and osteoporosis compared to the general population (Bultink and Lems, 2016; Rees et al., 2017; Mendoza-Pinto et al., 2018; Xia et al., 2019). Osteopenia is estimated to affect 25%–75% of SLE patients, while osteoporosis may occur in as many as 50% (Bultink and Lems, 2016). Moreover, abnormally low bone density has been observed even in very young SLE patients (Lim et al., 2011; Caetano et al., 2015). It is important to emphasize that osteoporotic fractures may occur in SLE patients even with normal bone density, as multiple coexisting factors simultaneously impair bone quality (Bultink and Lems, 2016; Buttgereit et al., 2024).

These factors include, first and foremost, the pharmacotherapy used in SLE. Secondly, lifestyle modifications recommended for SLE patients, such as avoiding UV radiation, may lead to vitamin D deficiencies without appropriate supplementation. Thirdly, disease-related factors such as chronic inflammation and hormonal imbalances also negatively affect bone density and quality (Islam et al., 2019; Zhu et al., 2021). Additionally, the development of complications associated with the disease can further complicate the situation. For example, lupus nephritis can impair calcium absorption from the intestines due to a lack of renal activation of vitamin D, and the frequently developing frailty syndrome increases the risk of falls (Zucchi et al., 2023; Schilirò et al., 2024).

Even in newly diagnosed, untreated SLE patients, a significantly higher prevalence of osteoporosis has been noted compared to the general population (Zhu et al., 2021). Additionally, lower BMD values and bone formation markers have been observed in these patients, while the levels of the bone resorption marker B-CTX were higher than in the gender-, age- and body mass index [BMI]-matched healthy control group and were positively correlated with disease activity. These observations confirm the significant impact of disease activity on bone resorption processes (Bultink and Lems, 2016; Buttgereit et al., 2024; Zhu et al., 2021). The increased prevalence of osteoporosis in SLE patients is also influenced by the fact that SLE occurs more frequently in women and that cytotoxic therapy induces premature menopause (Phang et al., 2018). A significant and well-known risk factor for osteoporosis in SLE patients is chronic glucocorticoid therapy, which is the most common cause of secondary osteoporosis in the general population (Beauvais et al., 2019).

The consequence of osteoporosis is fractures, which are also more commonly reported in SLE patients (Wang et al., 2016; Tedeschi et al., 2019). Studies have shown a significantly higher fracture risk in young SLE patients compared to a healthy control group (Wang et al., 2016).

Unfortunately, there are currently no detailed recommendations for osteoporosis prevention in SLE patients (Adami et al., 2019; Orsolini et al., 2020). The updated 2023 European League Against Rheumatism [EULAR] guidelines for lupus treatment are very general in this regard. As one of the overarching principles of lupus treatment, experts emphasize the role of nonpharmacological interventions aimed at promoting bone health (Fanouriakis et al., 2024). In a separate EULAR document dedicated to non-pharmacological treatment of lupus, the importance of bone health monitoring is highlighted, but specific actions to be taken are not detailed (Parodis et al., 2024). The only more specific and thus more clinically useful document is the American College of Rheumatology [ACR] guidelines on the prevention and treatment of secondary osteoporosis in patients receiving chronic glucocorticoid therapy. ACR experts emphasize the importance of: i) a diet and supplementation that ensures adequate calcium and vitamin D intake; ii) early initiation and regular assessment of fracture risk [using the FRAX calculator and densitometry] in patients chronically treated with glucocorticoids [GCS]: ≥2.5 mg/day prednisone or equivalent for more than 3 months; and iii) timely initiation of anti-osteoporotic therapy when indicated (Humphrey et al., 2023).

Given that osteoporosis prevention in patients with SLE may be underemphasized in clinical practice, this preliminary study aimed to assess: i) dietary and supplemental calcium intake; ii) frequency of vitamin D supplementation; iii) patient awareness of the increased osteoporosis risk in SLE; and iv) the extent of preventive measures implemented by healthcare professionals.

## Materials and methods

This was an observational cross-sectional study and was not registered as a clinical trial. The inclusion criteria for the study were an age of over 18 years and a diagnosis of SLE confirmed by a rheumatologist based on the EULAR/ACR criteria. Ethical approval was obtained from the Ethics Committee of the Medical University of Gdańsk (protocol number NKBBN/343/2018, approved on 10 October 2018), and all participants provided informed consent.

Out of 50 SLE patients, 28 consented to participate in the study. They were recruited consecutively, either in person or through a closed online community for people with SLE, from various rheumatology clinics across Poland. Data collection took place from December, 2022 to March, 2023.

Participants completed (either in person or remotely) the ADOS-Ca questionnaire, a validated tool in the Polish population for estimating calcium intake from the diet based on the frequency of consumption of various calcium-rich food groups (Szymelfejnik et al., 2006). The survey collected also information on whether the respondent:

1. Supplements calcium and in what dosage
2. Regularly supplements vitamin D, specifying the dosage and time of year
3. Uses UV protection creams on forearms and lower legs during summer sun exposure
4. Receives GCS, including the type of preparation, dosage, and duration of use
5. Is aware that SLE patients have a higher risk of developing osteoporosis
6. Has received education on osteoporosis prevention during medical consultations
7. Has ever had serum 25(OH)D levels assessed
8. Has ever had fracture risk assessed through bone densitometry
9. Has ever experienced low-energy fractures
10. Receives anti-osteoporotic medication

The recommended daily calcium intake was based on guidelines for the Polish population and other expert statement, set at 1,000 mg for women and men ≤50 years and 1,200 mg for women >50 years and men >65 years (Kanis et al., 2019; Wojtasik et al., 2020; Humphrey et al., 2023). The dosage of GCS other than prednisone was reported as a prednisone equivalent in mg/day. According to ACR guidelines, a high GCS dose was defined as ≥7.5 mg prednisone or equivalent per day (Humphrey et al., 2023).

A low-energy fracture was defined in the questionnaire as a fracture of the vertebral body, femur, forearm, humerus, or pelvis resulting from a fall from standing height or a spontaneous fracture.

### Statistical analysis

For numerical data, the Shapiro-Wilk test was used to assess normal distribution. The demographic characteristics were described as mean ± standard deviation (SD) for normally distributed variables and as median with interquartile range (Q1-Q3) for non-normally distributed variables. Categorical variables were presented as frequen-cies and percentages.

The Student’s t-test (or the Mann-Whitney U, when applicable) was used to com-pare means between two groups. For comparing more than two groups with non-normally distributed data, the Kruskal-Wallis one-way analysis of variance by ranks was used. The chi-square test (or Fisher’s exact test, when applicable) was used to analyze categorical data.

Statistical analysis of the collected data was performed using Statistica version 13.3 (Statsoft Polska). A significance level of p < 0.05 was adopted.

## Results

### Characteristics of the study group

In the study group, 96% of participants were female. The average age was 38.6 ± 10.1 years, with a BMI of 22.9 ± 3.0. The median (Q1-Q3) time since SLE diagnosis was 8.0 (2.5–11.0) years. Nearly half of the patients (43%) were aged ≥40 years. More than half of the group (57%) had been on glucocorticoid therapy for ≥3 months. The median GCS dose was 5.0 (5.0–8.75) mg prednisone equivalent per day, with a median treatment duration of 6.5 (2.0–13.5) years.

A high GCS dose, defined as ≥7.5 mg prednisone equivalent per day according to ACR guidelines, was administered to 21% of patients, including one individual who received ≥30 mg/day.

### Calcium intake from diet and supplementation

Based on the ADOS-Ca questionnaire most of the study population (22/28, 79%) did not meet the recommended dietary calcium intake. In a subgroup of GCS-treated, inadequate calcium intake from diet was observed in 81% of cases (13/16). The median dietary calcium intake in the entire SLE cohort was 540 (387–994) mg/day, with a similarly low median intake of 498 (369–984) mg/day in a subgroup of GCS-treated.

**FIGURE 1:**
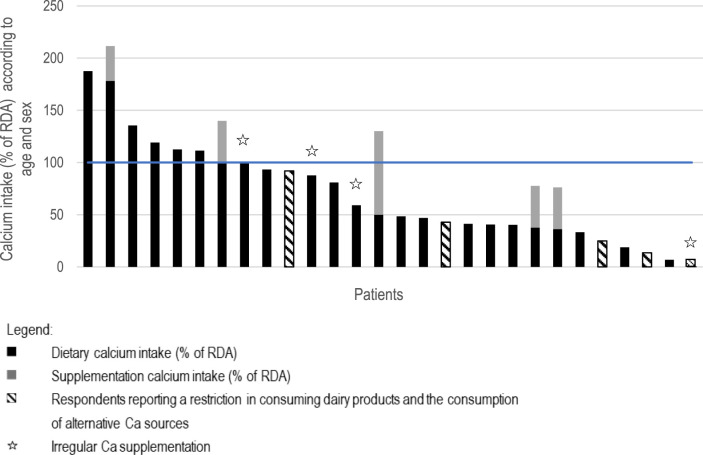
Individual Ca intake presented as percentage of Recommended Dietary Allowance depending on age and sex (RDA, blue line) from diet and supplementation according to the results of the ADOS-Ca questionnaire.

Only 18% of SLE patients (5/28) regularly supplemented calcium, all of whom belonged to the subgroup undergoing chronic GCS therapy. This represents only one third (5/16) of chronic GCS subgroup who supplemented calcium. When accounting for both dietary and supplemental calcium intake, the percentage of individuals meeting the recommended intake in the entire group increased from 21% to 29%. These results are presented in Table 1. The individual calcium intake from diet alone and in combination with supplementation in the entire study population is illustrated in Figure 1.

**TABLE 1:** Characteristics of the entire group with SLE and the subgroup treated chronically with GCS, and the results obtained.

Variable	All participants (N = 28)	Treated with GCS (N = 16)	P Treated vs. untreated with GCS
Age, years	38.6 ± 10.1	40.7 ± 10.9	0.46^a^
Participants ≥40 years old	12 (43%)	9 (56%)	0.48^b^
Women	27 (96%)	15 (94%)	>0.99^b^
BMI (kg/m^2^)	22.9 ± 3.0	23.5 ± 3.4	0.13^a^
Time since SLE diagnosis, years	8.0 (2.5–11.0)	10.0 (3.0–17.0)	0.12^c^
Time treated with GCS, years	−	6.5 (2.0–13.5)	_
Current dose of GCS, mg prednisone/day	−	5.0 (5.0–8.75)	_
Dietary calcium intake, mg/day	540 (387–994)	498 (369–984)	0.51^c^
Meeting recommended dietary Ca intake	6 (21%)	3 (19%)	>0.99^b^
Ca intake from diet and supplementation, mg/day	769 (404–1,119)	769 (402–1,152)	>0.99^c^
Meeting recommended Ca intake from diet and supplementation combined	8 (29%)	5 (31%)	>0.99^b^
Regularly supplementing vitamin D	22 (79%)	11 (69%)	0.78^b^

Abbreviations: Ca - Calcium, GCS, Glucocorticosteroids; SLE, Systemic Lupus Erythematosus.

^a^ Student’s t test.

^b^ Fisher’s exact test.

^c^ U Mann-Whitney test.

### Vitamin D supplementation and serum 25(OH)D concentration assessment

Regular vitamin D supplementation was reported by the majority (79%) of the SLE patients. The most common supplementation doses were 2000 IU (46%) and 4000 IU (42%). Among respondents who reported using UV protection on their forearms and lower legs during summer sun exposure (20/28), a larger part of the cohort (15 persons) confirmed year-round vitamin D supplementation. Similar results were obtained in the subgroup of GCS users, where 11 patients (69%) reported regular vitamin D supplementation, and among those using UV protection (13/16, 81%), only 9 persons confirmed year-round vitamin D supplementation.

Daily calcium intake and the frequency of vitamin D supplementation did not differ across subgroups of participants stratified by age, BMI, or disease duration (data presented in Table 2).

**TABLE 2:** Daily dietary calcium intake and frequency of regular vitamin D supplementation in subgroups of SLE patients categorized according to age, BMI and disease duration.

Variable	Dietary Ca intake mg/day	p	Vit supplementation N (%)	p
Age, years ≥40 <40	898 (219–1,056) 497 (404–780)	0.94^a^	8/12 (67%) 14/16 (87%)	0.77^b^
BMI, kg/m^2^ ≥25 <25	733 (362–1,355) 540 (399–990)	0.63^a^	4/6 (67%) 18/22 (82%)	>0.99^b^
Time since SLE diagnosis, years <4 4–10 >10	926 (188–999) 875 (500–1,114) 406 (399–495)	0.35^c^	9/10 (90%) 6/9 (67%) 7/9 (78%)	0.46^d^

Abbreviations: Ca - Calcium, SLE, Systemic Lupus Erythematosus.

^a^ U Mann-Whitney test.

^b^ Fisher’s exact test.

^c^ Kruskal-Wallis one-way analysis of variance by ranks.

^d^ Chi-square test.

Serum 25(OH)D levels were assessed at least once in 46% of the entire group (13/28) and in 10 patients of the of GCS using subgroup.

### Patient awareness of increased osteoporosis risk and preventive actions taken by medical personnel

Approximately 57% of patients acknowledged being aware of the increased osteoporosis risk associated with SLE. Most patients (86%) were aware of the indications for vitamin D supplementation in SLE. However, only 32% of SLE patients reported receiving education about other osteoporosis prevention methods during medical consultations.

Bone densitometry had been performed for only one patients from the non-GCS users and six GCS-treated. None of the SLE patients ≥40 years of age reported having their fracture risk assessed using the FRAX calculator.

The prevalence of osteoporosis in the study group, based on self-reported diagnoses, was 11% (3 patients). Only one patient with a reported diagnosis of osteoporosis was receiving anti-osteoporotic treatment (bisphosphonates). Additionally, 2 patients (8% of the group) who had not been previously diagnosed with osteoporosis reported having sustained low-energy fractures.

## Discussion

### Importance of a comprehensive approach to bone health in SLE

The extremely wide range of osteopenia and osteoporosis prevalence among patients with SLE suggests that different studies have not applied consistent diagnostic criteria and that the studied populations were likely heterogeneous. Nevertheless, it is undeniable that osteoporosis is a serious and burdensome condition, often undiagnosed and/or untreated (Bultink and Lems, 2016; Singer et al., 2023).

The updated EULAR guidelines for lupus therapy emphasize the importance bone health monitoring but do not specify preventive recommendations (Fanouriakis et al., 2024; Parodis et al., 2024). Due to avoidance of UV radiation, vitamin D supplementation is advised, alongside a balanced diet and other lifestyle modifications, f.e. physical activity (Fanouriakis et al., 2024; Parodis et al., 2024). Experts also underscore the significance of multidisciplinary care to improve the quality of life for lupus patients. In this context, individual dietary components, such as calcium, should not be analyzed in isolation but rather as part of broader dietary patterns that influence bone metabolism, muscle mass, and the risk of falls.

The aim of the present study was not to demonstrate a causal relationship between calcium intake and BMD or fracture risk, but rather to identify a potential, modifiable risk factor relevant to everyday clinical practice.

### Dietary patterns and calcium intake

While evaluating the effectiveness of vitamin D supplementation is relatively straightforward, monitoring calcium intake is more complex. It is rarely assessed, and even osteoporosis treatment guidelines do not propose systematic solutions for managing this issue (Cianferotti et al., 2024). Dietary calcium intake can be assessed using dietary recall methods, but these are time-consuming. A more efficient and reliable alternative may be food frequency questionnaires, such as the ADOS-Ca, which allows for a quick and relatively accurate estimation of daily calcium intake and identification of patients at risk of deficiency (Szymelfejnik et al., 2006).

Using ADOS-Ca, we found that the majority of SLE patients (79%) did not consume the recommended amount of calcium from their diet. Similarly, this behavior was poor in the subgroup taking GCS (81%). The median calcium intake for the entire study group was 540 mg/day, and in the subgroup taking GCS, it was 498 mg. Our results relate to reports by authors who previously assessed calcium intake among SLE patients, and their observations align with ours. In a larger SLE patient group from Brazil, daily calcium intake assessed by a 1-day interview was 404.5 mg, and most respondents did not meet the recommended dietary intake (Borges et al., 2012). In SLE patients from Spain, the results looked slightly better. Calcium intake according to a 1-day interview was 821 mg/day, yet 62.5% of respondents did not meet the recommended intake (Pocovi-Gerardino et al., 2018).

Both our findings and those reported by the cited authors may indicate that low calcium intake is common in the studied populations of patients with SLE and appears to reflect long-standing, suboptimal dietary patterns rather than merely a lack of supplementation.

It should be also noted that our observed calcium intake among Polish SLE patients does not deviate from the average intake of this macronutrient in the Polish general population. This may suggest that patients with SLE do not appear to offset their increased risk of bone-related complications through dietary modification. According to current data from the Polish Institute of Food and Nutrition in Warsaw, dietary calcium supply was 598 mg/day (Wojtasik et al., 2020). Slightly higher values were obtained in a study of a representative group of older Poles (45–64 years old) conducted in 2010–2011 - in males: 660.6 mg/day and in females: 703.6 mg/day (Ilow et al., 2011).

It is likely that in other European countries such as Spain, Italy, Austria, France, Germany, Belgium, and the Netherlands, dietary calcium supply may be higher - between 765 and 1,102 mg/day (Balk et al., 2017). However, it is important to consider that current sociocultural conditions, especially among young people, promote vegan diets, in which achieving the daily recommended amount of calcium is challenging without the supervision of a dietitian (Clarys et al., 2014; Craig et al., 2021).

It should be noted that evidence from some studies suggests no association between calcium intake and bone mineral density or fracture incidence, both in the general population (Skowrońska-Jóźwiak, 2014; Tai et al., 2015) and among patients with SLE (Chong et al., 2007). This is likely because low calcium intake represents only one of many factors influencing fracture risk, alongside overall diet quality, vitamin D status, level of physical activity, menopausal status, ethnicity, as well as comorbidities and therapeutic interventions. It is also worth noting that interventions focused on a single nutrient do not alter overall dietary patterns toward a health-promoting profile and, consequently, may not affect key determinants of bone health. Consequently, access to a registered dietitian should be probabely an integral part of multidisciplinary and comprehensive care for patients with SLE.

### Calcium supplementation – a complementary rather than dominant role

Calcium supplementation can complement dietary intake to reach the level recommended for bone health.

However, only 31% (5/16) of the subgroup receiving chronic GCS utilized regular calcium supplementation, although according to ACR experts, most such patients are indicated for calcium supplementation. Moreover, this supplementation was not effective for everyone. Only two individuals out of five optimized their total calcium intake to the recommended level. It was also observed that one of the individuals taking additional calcium already received an age-appropriate amount from the diet, and as a result of additional supplementation, slightly exceeded the upper tolerated intake level for adults (2,500 mg/day) (Wojtasik et al., 2020). However, the remaining two participants, despite additional calcium supply, did not meet the age-specific intake norms (data from Figure 1).

Summarizing, calcium supplementation in our studied group was infrequent and often inadequately matched to actual dietary intake, leading both to persistent deficiencies and, in some cases, to potential exceedance of the upper tolerable intake level.

These observations also suggest that within multidisciplinary care for lupus patients, there should be a role for a dietitian. This healthcare professional could provide group education on dietary sources of calcium, factors influencing its absorption, and methods for assessing calcium intake, such as the ADOS-Ca questionnaire. For patients suspected of having low calcium intake, the dietitian could help in optimizing dietary consumption of this macronutrient and/or setting a tailored supplemental dose of a well-tolerated calcium preparation.

### Vitamin D supplementation as part of a broader preventive strategy

The study revealed that the majority of our group of Polish SLE patients (nearly 80%) were aware of the importance of regular vitamin D supplementation and strive to adhere to this practice. Vitamin D concentration is subject to changes associated with seasonal rhythms (Osredkar et al., 2024). However, only 75% of those who reported using UV protection during the summer confirmed year-round vitamin D supplementation. This finding suggests a potential risk of vitamin D deficiency in this group. Unfortunately, only about half of the participants reported having their serum 25(OH)D levels assessed, which is desirable to confirm optimal supplementation dosages. In a similar small pilot study (N = 39) that evaluated the average serum vitamin D levels among SLE patients, the mean level was 30.95 ng/mL, with an average supplementation dose of 1398 IU/day. As in the present study, 80% of the participants were supplementing vitamin D. Serum 25(OH)D levels below 30 ng/mL were observed in 43.8% of the group (Runowska et al., 2021). Other studies have reported vitamin D deficiency in more than 65% of lupus patients (Salman-Monte et al., 2017).

More detailed osteoporosis prevention guidelines are available for patients receiving GCS therapy (Humphrey et al., 2023). ACR experts recommend optimizing calcium and vitamin D intake through diet and supplementation for all patients on chronic GCS, along with resistance exercises, avoiding smoking and excessive alcohol consumption. Additionally, all patients are advised to monitor serum vitamin D levels to maintain concentrations of 30–50 ng/mL. In our study, the high proportion of patients reporting vitamin D supplementation suggests increased awareness of this factor; however, the lack of systematic monitoring of serum 25(OH)D levels limits the ability to assess the effectiveness of this intervention. It is worth adding that authors from Saudi Arabia demonstrated that 6-month supplementation of calcium and vitamin D in SLE patients with initially confirmed vitamin D deficiency led to a significant increase in BMD (Al-Kushi et al., 2018). Thus, vitamin D, similarly to calcium, should be considered as part of a comprehensive preventive strategy encompassing diet, physical activity, and fracture risk assessment.

### The gap between recommendations and clinical practice

The low frequency of bone densitometry, lack of use of fracture risk assessment tools (FRAX), and infrequent initiation of anti-osteoporotic therapy indicate a significant gap in osteoporosis prevention among patients with SLE. The ACR experts suggest conducting a fracture risk assessment for all patients taking GCS at doses ≥2.5 mg/day for 3 months or more (Humphrey et al., 2023). In the present study, it was shown that only 37% of patients taking GCS (with a median time from SLE diagnosis of 10 years) had undergone densitometry. None declared that their 10-year fracture risk was assessed using the FRAX calculator. It cannot be excluded that patients were unaware of what the FRAX risk assessment entails or were not informed about its execution by their physician.

It is also concerning that only one patient from the entire study group received antiosteoporotic treatment, although three patients declared having osteoporosis (11% of the study group), and two others (8% of the group) confirmed experiencing low-energy fractures. It is also noteworthy that in our group, a high GCS dose (≥7.5 mg/day), significantly increasing the risk of fractures, was used by 21% of the patients, and one individual took ≥30 mg/day, a dose according to ACR guidelines that qualifies a patient for a very high fracture risk group (Humphrey et al., 2023).

It is interesting to note that other authors have obtained equally concerning results regarding the implementation in clinical practice of guidelines for the prevention and treatment of poststeroid osteoporosis in SLE patients. In a study published in 2020 assessing the implementation of ACR 2017 guidelines regarding vitamin D supplementation and bisphosphonate treatment, it was found that only about 60% of SLE patients taking chronic GCS received vitamin D supplementation, and bisphosphonate treatment was initiated in 21% of patients, while 36% of the participants had indications for treatment based on FRAX assessment (Sapkota et al., 2020). Therefore, there is likely a significant gap in care for SLE patients, meaning a substantial number of individuals are at risk of osteoporotic fractures but are not receiving either prophylaxis or treatment (Compston, 2020). These findings confirm the need for a multidisciplinary approach in which the assessment of dietary patterns and nutritional education constitute an integral component of patient care.

### Limitations and strengths

Our study has several significant limitations. The relatively small number of participants constitutes the most important limitation of the present study and may restrict the generalizability of the findings to the broader population of patients with systemic lupus erythematosus. Another limitation is cross-sectional nature of the study. Since many patients declined to participate in the study, selection bias is possible. Moreover, the survey data were collected based on patient declarations, thus relying on their knowledge of their health status and treatment course, which may be associated with recall bias. There was also no possibility to assess the so-called cumulative dose of GCS. Therefore, it is possible that more patients would be classified into the very high fracture risk group. Consequently, the observed results should be interpreted with caution.

Despite these limitations, our results can be useful for clinical practice, indicating an area of suboptimal care for SLE patients, namely osteoporosis prevention. Equally important is the fact that implementing education on the increased osteoporosis risk in this group of patients and supporting them in optimizing calcium intake are both reasonable and entirely risk-free approaches in clinical practice.

### Conclusions

These findings draw attention to the problem of insufficient calcium supply in the group of patients with SLE at increased risk of decreased bone mineral density and the need to adjust the supplemental calcium dose to its intake to optimize supply in accordance with prevention and treatment recommendations for osteoporosis. Low calcium intake from diet and supplements is common among patients with SLE and may constitute one of several modifiable factors contributing to adverse bone outcomes; however, it should not be regarded as the dominant risk factor. The findings further indicate the need for closer collaboration with registered dietitians in the care of patients with SLE and for greater emphasis on preventive measures by healthcare professionals, as well as increased patient awareness regarding bone health.

## Data Availability

The raw data supporting the conclusions of this article will be made available by the authors, without undue reservation.
